# Microbial regulation of microRNA expression in the amygdala and prefrontal cortex

**DOI:** 10.1186/s40168-017-0321-3

**Published:** 2017-08-25

**Authors:** Alan E. Hoban, Roman M. Stilling, Gerard M. Moloney, Rachel D. Moloney, Fergus Shanahan, Timothy G. Dinan, John F. Cryan, Gerard Clarke

**Affiliations:** 1 APC Microbiome Institute, University College Cork, Cork City, Ireland; 2 Department of Anatomy and Neuroscience, University College Cork, Cork City, Ireland; 3 Department of Psychiatry and Neurobehavioural Science, Biosciences Institute, University College Cork, Room 1.15, College Road, Cork City, Ireland

**Keywords:** Amygdala, Prefrontal cortex, Microbiome-gut-brain axis, MicroRNAs, Germ-free, Antibiotics, miR-206-3p

## Abstract

**Background:**

There is growing evidence for a role of the gut microbiome in shaping behaviour relevant to many psychiatric and neurological disorders. Preclinical studies using germ-free (GF) animals have been essential in contributing to our current understanding of the potential importance of the host microbiome for neurodevelopment and behaviour. In particular, it has been repeatedly demonstrated that manipulation of the gut microbiome modulates anxiety-like behaviours. The neural circuits that underlie anxiety- and fear-related behaviours are complex and heavily depend on functional communication between the amygdala and prefrontal cortex (PFC). Previously, we have shown that the transcriptional networks within the amygdala and PFC of GF mice are altered. MicroRNAs (miRNAs) act through translational repression to control gene translation and have also been implicated in anxiety-like behaviours. However, it is unknown whether these features of host post-transcriptional machinery are also recruited by the gut microbiome to exert control over CNS transcriptional networks.

**Results:**

We conducted Illumina® next-generation sequencing (NGS) in the amygdala and PFC of conventional, GF and germ-free colonized mice (exGF). We found a large proportion of miRNAs to be dysregulated in GF animals in both brain regions (103 in the amygdala and 31 in the PFC). Additionally, colonization of GF mice normalized some of the noted alterations. Next, we used a complementary approach to GF by manipulating the adult rat microbiome with an antibiotic cocktail to deplete the gut microbiota and found that this strategy also impacted the expression of relevant miRNAs.

**Conclusion:**

These results suggest that the microbiome is necessary for appropriate regulation of miRNA expression in brain regions implicated in anxiety-like behaviours.

**Electronic supplementary material:**

The online version of this article (doi:10.1186/s40168-017-0321-3) contains supplementary material, which is available to authorized users.

## Background

One of the most exciting findings in the past decade in relation to psychiatric disorders has been the discovery that the gut microbiota may act as a key regulator of the brain and behaviour [1–3]. Proof-of-principal experiments using microbiota-deficient or germ-free (GF) rodents have been extremely useful in investigating the impact of the absence of the gut microbiota on brain development and behaviour [4], especially regarding the ability of the gut microbiota to influence normal anxiety and fear behaviours [5–7]. GF animals not only display changes in basal anxiety but also have deficits in sociability, cognition and increased depressive-like behaviours [8–10]. Some studies that use antibiotic exposure in order to deplete the gut microbiota have also shown similarly altered behavioural phenotypes [11–13]. Preclinical evidence also highlights that certain probiotics and prebiotics have anxiolytic-like activity [14, 15] which highlights the potential of microbiota-mediated therapeutic approaches for treating anxiety-related disorders.

The most reproducible finding with regard to behavioural effects in rodent models of microbial disturbances is a marked influence on anxiety-related behaviours [4]. The neural systems that govern such behaviours are complex, with an array of brain regions that interconnect in order to process emotional stimuli and allow for appropriate responses [16]. Specifically, the amygdala and PFC are key loci that control anxiety and the response to fearful stimuli [17]. Both regions display abnormalities in GF animals including hypermyelination in the PFC [18], altered morphology of the amygdala [19] and impaired amygdala-dependent fear memory recall [7]. A large body of evidence implicates dysfunction of the neural circuits connecting these two brain regions in the pathophysiology of fear- and anxiety-like disorders [20, 21].

Over the past few years, attempts to understand the mechanisms underlying psychiatric disorders have focused attention on the role of miRNAs [22]. miRNAs are an expanding class of endogenous small non-coding RNAs, which function as post-transcriptional regulators of gene expression, primarily through translational repression. It has been demonstrated that brain-specific miRNA candidates within the amygdala and PFC regulate anxiety- and fear-related behaviours in mice [23–26].

We have previously shown that the absence of microbial exposure throughout life results in altered transcriptional regulation in both the amygdala and PFC [18, 27]. We therefore hypothesized that alterations in miRNA expression may be associated with some of the noted molecular changes in these animals. To this end, we used a number of complementary approaches. Firstly, we conducted unbiased Illumina® NGS for miRNAs in the amygdala and PFC of conventionally raised (CON), GF and colonized GF mice (exGF) to investigate whether miRNA expression can be influenced by the gut microbiota. To delineate specific miRNAs that may be influenced by the gut microbiota during adulthood following normal assembly patterns in early life, we then examined miRNA expression in the central nervous system (CNS) of adult rodents following sustained microbiota knockdown with antibiotics. We hypothesized that there would be extensive reorganization of miRNA expression in these two key brain regions (amygdala and PFC) of microbiota-deficient rodents.

## Methods

### Experimental design

Illumina® NGS was conducted in GF mice to determine whether the absence of microbes throughout life resulted in altered miRNA expression in the amygdala and PFC. Once validation of sequencing in GF animals was carried out, we then examined whether these miRNAs identified in GF mice were affected in other strategies known to manipulate the gut microbiota. Microbiota (bacteria)-deficient GF mice were compared to rodents, the latter with their digestive tract/gut bacteria controlled by antibiotics to delineate/validate common gut bacteria that influence brain-specific miRNAs. The rationale for using this complementary approach to GF was to ensure our findings were not species specific and to thus identify a lead candidate miRNA under the influence of the gut microbiota in both mice and rats.

### Animals

#### Germ free

Swiss Webster GF and CON breeding pairs were obtained from Taconic (Germantown, NY, USA) with F_1_-generation offspring used in all experiments. GF mice were housed in gnotobiotic flexible-film isolators ranging from two to four mice per cage kept on a strict 12-h light/dark cycle. exGF mice were all initially raised within the GF isolators until post-natal day 21 where they were removed and housed in standard animal units next to CON mice in order to allow for efficient colonization by environmental microbes [28]. exGF mice were initially put in cages with dirty bedding from CON; as mice are coprophagic, this allows for efficient colonization [28]. CON mice were housed in controlled conditions with regulated temperature (20–21 °C) and humidity (55–60%) with two to four mice per cage on the same 12-h light/dark cycle as GF mice. All mice, CON, GF and exGF received the same autoclaved, pelleted diet (Special Diet Services, product code 801010). All experiments were conducted in accordance with European Directive 2010/63/EU. Approval by the Animal Experimentation Ethics Committee of University College Cork was obtained before commencement of all animal-related experiments. Both the conventional and GF facilities adhere to the same animal care guidelines in terms of temperature, humidity and noise levels.

#### Antibiotic treatment

Adult male Sprague Dawley rats (*n* = 10/group) were housed five per cage in standard rat cages in our animal housing facility under a strict 12-h light/dark cycle. Both antibiotic-treated and vehicle-treated rats received the same autoclaved diet (Teklad Global 18% Protein Rodent Diet, product code 2018S). To deplete the gut microbiota, rats were treated with a cocktail of antibiotics for a total of 13 weeks; animals were 9 weeks old before antibiotic exposure. The antibiotic cocktail consisted of ampicillin (1 g/L), vancomycin (500 mg/L), ciprofloxacin HCL (20 mg/L), imipenem (250 mg/L) and metrondiazole (1 g/L) in autoclaved water. This was changed every 3 days as previously described to deplete the gut bacteria [29, 30]. Control animals received autoclaved water without any antibiotics which was also changed every 3 days. Additional details on experimental design and neurochemical and behavioural consequences of chronic gut microbiota depletion can be found in our previous publication [30].

### RNA extraction

The amygdala and PFC was dissected as previously described [18, 27]. Following manufacturer’s protocol, a mirVana™ miRNA kit (Ambion/Life Technologies) was used to extract total RNA from germ-free and antibiotic-treated animals. A NanoDrop 1000 (Thermo Scientific) and a Bioanalyzer were used to determine concentration and RNA integrity number (RIN). Only GF samples, conventional and exGF animals were randomly pooled within each group by combining equal amounts of RNA from two to three animals resulting in a final sample group of four. Experimental pooling for GF samples was carried out according to a previously published protocol [18].

### MicroRNA sequencing

Library preparation and next-generation sequencing was conducted on all samples. Two hundred nanograms of total RNA was converted into miRNA NGS libraries using NEBNEXT library generation kit (New England Biolabs Inc.) according to manufacturer’s instructions. Each individual RNA sample was converted to complementary DNA (cDNA) which was pre-amplified. After 15 cycles pre-PCR, the libraries were purified on QiaQuick columns and the insert efficiency evaluated by the Bioanalyzer 2100. Based on the quality of the inserts and the concentration measurements, the libraries were pooled. Library pools were quantified by qPCR and used to generate clusters on the surface of a flowcell before sequencing. A total of 12 samples were sent for Illumina® NGS for miRNAs to Exiqon (Denmark) to determine changes in miRNA expression profiles (GF study animals only). Annotation of the obtained sequences was performed using the reference annotation miRbase 20 (http://www.mirbase.org/). miRNA sequencing was performed using the NextSeq500 with 50 bd single-end read sequencing cycles. Expression levels of individual miRNAs are measured as tags per million (TPM).

### cDNA synthesis and quantitative real-time PCR (qRT-PCR)

RNA was reverse transcribed using TaqMan® MicroRNA Reverse Transcription Kit (Applied Biosystems) for individual miRNAs in a G-storm thermocycler (G-storm, Surrey, UK). Using AB7300 system (Applied Biosystems) and TaqMan Gene Expression Assays (Additional file 1: Table S1) for individual miRNAs, expression levels were determined within the amygdala and prefrontal cortex. Each transcript value was averaged from triplicates per experimental condition. All average values were normalized to expression levels of the housekeeper gene *U6*, a small nuclear RNA, for each experimental condition. Fold change in gene expression was normalized against expression levels in naïve CON mice. qRT-PCR validation was conducted on all individual samples used to construct the pooled sequencing samples: germ-free (*n* = 12/group) and antibiotic (*n* = 10/group).

### mRNA target predictions

The miRwalk (http://zmf.umm.uni-heidelberg.de/apps/zmf/mirwalk2/) prediction database was used to identify predicted and validated targets for the miRNAs we found significantly dysregulated due to absence of bacterial exposure (GF vs. CON). Using miRwalk, we listed all predicted and validated messenger RNA (mRNA) targets for all differentially regulated miRNA in both the amygdala and prefrontal cortex. We selected for miRwalk to include predicted mRNA targets from four different prediction databases which included TargetSacn, miRanda, miRDB and miRwalk. Criteria for prediction included a minimal seed length of seven base pairs and a *P* value < 0.05 was used as a cut-off. For further analysis, we only chose predicted targets that occurred in three or more prediction algorithms.

### Functional classification of predicted miRNA targets

Predicted mRNA targets of differentially regulated miRNAs in GF mice compared to CON were analysed for enrichment of Gene Ontology (GO) terms and Kyoto Encyclopedia of Genes and Genomes (KEGG) pathways using the DAVID Bioinformatic Resources (v6.8). Lists of predicted targets were generated for any miRNA that fell under the selection criteria, and they were used to determine functionally enriched pathways that these miRNAs are predicted to be implicated in. FDR *P* adjusted value of 0.1 was used as the significance cut-off as per previous publications [7, 18, 27].

### mRNA interaction analysis

To find the correlation between differential expression of miRNAs and their target mRNAs, a series of bioinformatic analyses were performed. miRNAs are usually negatively correlated with their targeted mRNAs, except in some cases where translation might be enhanced [31]. In order to identify potential miRNA-regulated target genes in GF mice, the datasets of differentially expressed miRNA and mRNA transcripts from our previous publications [18, 27] were integrated. We set the following criteria for potential predicted targets. The target mRNAs and miRNAs should be simultaneously and reversely changed in our group comparisons. The target mRNAs should be predicted by miRNA from at least three different prediction software. The complement miRNA targets predicted were compared with those differentially regulated genes (DEGs) from our mRNA sequencing to detect overlap. For this analysis, we only focused on interactions with validated miRNAs in the amygdala. Since myelination was the strongest representation at the transcriptional level [18], we investigated whether any down-regulated miRNAs in the PFC were predicted to target upregulated myelin-related genes.

### Statistics

NGS differential expression analysis used the EdgeR statistical software package (Bioconductor, http://bioconductor.org/). Differential expression analysis investigates the relative change in expression (i.e. counts) between different samples. *P* values for significantly expressed miRNAs are estimated by an exact test on the negative binomial distribution. Data is presented with raw *P* value, Benjamini-Hochberg FDR correction, and corrected *P* value as well as the average read values per group **(**Additional file 2: Table S2). For quantification via qRT-PCR, gene expression was calculated using the 2^−ΔΔCt^ formula [32]. This value was then normalized to the control group to calculate fold change. One-way ANOVA, or non-parametric equivalent as appropriate, was used for gene expression analysis for GF comparisons. Post hoc analysis was carried out using Fisher’s Least Significant Difference (LSD) post hoc test. For antibiotic treatment, a Student *t* test was carried out between groups to determine significance. A *P* value < 0.05 was considered statistically significant.

## Results

### Altered miRNA expression profile in the amygdala and prefrontal cortex of germ-free mice

**Fig. 1:**
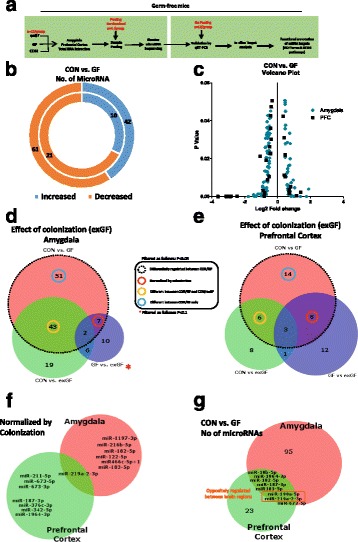
GF male mice display dysregulated network of miRNA expression in the amygdala and PFC. **a** Schematic representation of experimental design. **b** Donut plot representing the number of increased and decreased miRNA in the amygdala (*outer plot*) and PFC (*inner plot*) when comparing CON vs GF mice. **c** Volcano plot representing fold change against significance (*P* < 0.05) between CON and GF mice in the amygdala and PFC. **d** Venn diagram reporting overlapping differentially regulated miRNA between all three experimental groups in the amygdala representing the effect of colonization of GF mice on miRNAs. **e** Represents the impact of colonization of GF mice in the PFC. **f** Number of miRNAs by name that are normalized by colonization and common in both brain regions. **g** Number of microRNAs that are commonly dysregulated in both brain regions. *Highlighted in red* are those that are oppositely regulated in both brain regions

We conducted unbiased Illumina® NGS for miRNAs on extracted total RNA enriched for miRNA from the amygdala and PFC of male CON mice, GF mice and exGF mice (Fig. 1a). Initial analysis between CON and GF mice revealed a large number of miRNAs in the amygdala that appeared to have altered expression levels. In total, we found that 103 (61 downregulated and 42 upregulated) miRNAs showed changes with a *P* value of < 0.05 (Fig. 1b and Additional file 3: Figure S1a, b). In the PFC, we also found a number of differentially regulated miRNAs, though to a lesser extent than in the amygdala. A total of 31 miRNAs (21 downregulated, 10 upregulated) had altered expression in the PFC (Fig. 1b, Additional file 3: Figure S1c, d). All miRNAs differentially regulated in the amygdala and PFC had a fold change higher than 1.2 and changes were found across a large range of abundances (Log2 TPM) (Fig. 1c). When GF mice were colonized (exGF), we found that a proportion of differentially regulated miRNAs exhibited normalized expression levels, similar to the CON group. A total of 7 miRNAs in the amygdala and 8 miRNAs in the PFC were significantly normalized when overlapping their expression levels with CON vs GF comparison and GF vs exGF (red circle) (Fig. 1d–f). When we overlapped miRNAs that were normalized in both brain regions, we found that miR-219a-2-3p was differentially regulated in the amygdala and PFC of GF mice (Fig. 1f). Out of the miRNAs that were commonly dysregulated in both regions, miR-219a-2-3p, together with miR-190a-5p, was oppositely regulated between both regions (decreased in amygdala/increased in PFC) (Fig. 1g). Additional file 2: Table S2 lists all differentially regulated miRNAs.

### qRT-PCR validation confirms differentially regulated miRNAs in germ-free mice

**Fig. 2:**
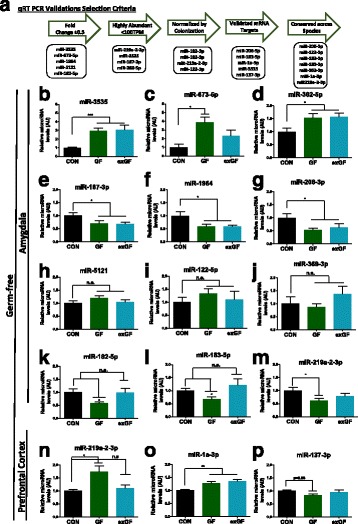
qRT-PCR validations of miRNA seq data within the amygdala and PFC of male GF mice. **a** Criteria for miRNA selection for validation via qRT-PCR. **b**–**m** qRT-PCR of miRNAs reported to be dysregulated in the amygdala of GF mice. **n**–**p** qRT-PCR validations of reported altered microRNA in the PFC. Bar graphs represent average values in 12 mice per group after *U6* normalization relative to average control levels. Fold changes is in comparison with CON group. Data graphed as ± SEM (*P* < 0.05*; *P* < 0.01**; *P* < 0.001***)

As our sequencing revealed a substantially large number of miRNAs, for validation purposes, we selected miRNAs that fulfilled specific criteria (Fig. 2a): high fold change, highly expressed (based on TPM), normalized by colonization, validated mRNA targets with associations in brain or behaviour research, and conservation across rodents and humans (Additional file 4: Table S3). We confirmed several miRNAs to be dysregulated in GF mice as indicated by Illumina® sequencing in both the amygdala and PFC (Fig. 2b–p). We demonstrated the presence of miRNAs that had a large fold increase (miR-3535, miR-673-5p) or decrease (miR-182-5p, miR-1964, miR-206-3p), that were normalized by colonization (miR-219a-2-3p (PFC), miR-182-5p, miR-183-5p (amygdala)) and that are known to be implicated in influencing anxiety levels and expression of neurotrophins such as brain-derived neurotrophic factor (BDNF) (miR-183-5p, miR-206-3p) [33, 34].

### Enrichment of predicted mRNA targets for all differentially regulated miRNA suggests a role in neurodevelopment

**Fig. 3:**
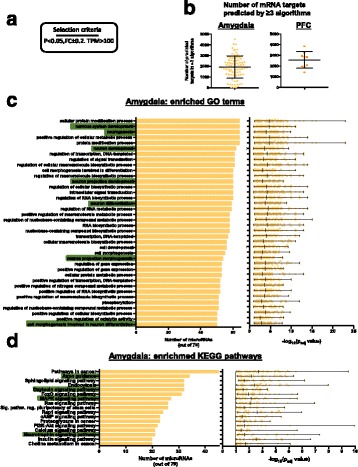
Functional enrichment analysis of predicted mRNA targets of differentially regulated miRNAs in the amygdala. **a** Selection criteria for miRNAs. **b** Number of mRNA targets predicted by miRwalk that appeared in more than three prediction algorithms. **c** Number of miRNAs (out of 79) where its predicted targets are enriched for GO terms. *Highlighted in green* are GO terms of interest implicating miRNA in neurodevelopment. **d** KEGG pathways that are predicted to be enriched in the amygdala based on the mRNA targets of all differentially regulated miRNAs in GF mice. Graphs depict the number of miRNAs that have predicted mRNA targets that are fall into specific GO terms and KEGG pathways. Scatter plot depicts how significant individual miRNAs are enriched for a specific GO term or KEGG pathway

To elucidate the potential downstream gene networks relevant in the amygdala and PFC that may be under the influence of miRNAs in GF animals, we listed all potential mRNA targets for individual miRNAs that were significantly differentially regulated between CON and GF and that had a PCR-detectable read count (TPM > 100) in both brain regions (79 miRs in amygdala and 9 in PFC). We listed all targets that appeared in more than 3 target-prediction algorithms (miRWalk) and examined GO terms and KEGG pathways. In the amygdala, we found an enrichment in GO terms for neuronal development including neurogenesis, neuron projection development, differentiation and morphogenesis (Fig. 3c, highlighted). KEGG pathway analysis of predicted mRNA targets featured enrichment for axon guidance, MAPK, oxytocin and neurotrophic signalling pathways (Fig. 3d). While the individual miRNAs were largely different, within the PFC highly similar GO terms and KEGG pathways were noted (Additional file 5: Figure S2a–c). In line with a higher number of differentially expressed miRNAs in the amygdala, several unique GO terms and pathways were found enriched in this region, with only few pathways enriched in a region-specific manner in the PFC (Additional file 5: Figure S2C).

To further highlight the potential regulatory role of these miRNAs at the transcriptional level, we listed all predicted targets (< 3 prediction algorithms) of qRT-PCR-validated miRNAs in the amygdala and overlapped them with our previously published data on the amygdala transcriptome of GF mice [27]. We found a predicted interaction with dysregulated miRNAs in the amygdala that are predicted to target mRNA transcripts that are also altered in GF mice (Additional file 6: Table S4). Since hypermyelination was the strongest finding in the PFC [18], we focused on miRNAs in the PFC that are predicted to target myelin-related genes (Additional file 7: Table S5).

### Antibiotic exposure alters expression of miRNAs in the amygdala and PFC in rats

**Fig. 4:**
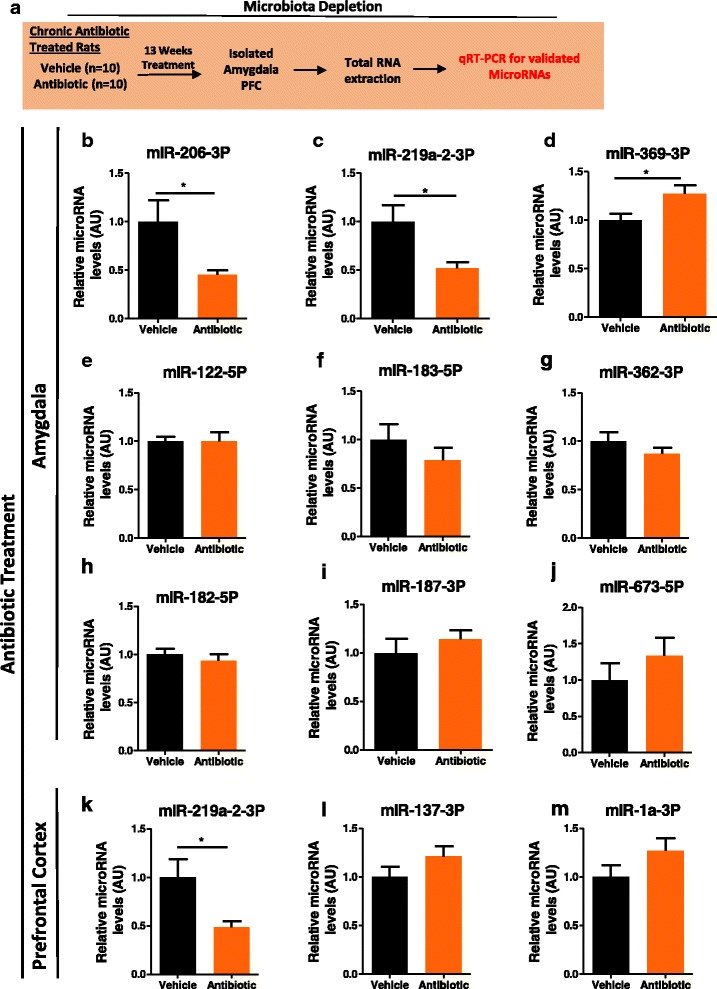
Antibiotic exposure in male rats alters miRNA expression in the amygdala and PFC. **a** Schematic of experimental approach. **b**–**j** qRT-PCR of miRNA expression in the amygdala of antibiotic-treated rats as compared to vehicle treated. **k**–**m** Expression levels of miRNA in the PFC. Bar graphs represent average values in 10 mice per group after *U6* normalization relative to average vehicle control-treated rats. Fold changes are compared to vehicle group. Data graphed as ± SEM. (*P* < 0.05*)

Antibiotic exposure is a useful strategy for directly depleting the gut microbiota in rodents [11, 13]. We investigated whether miRNAs that were dysregulated in GF mice had altered expression after antibiotic exposure. We found a number of miRNAs to be altered in the amygdala and PFC of rats after chronic long-term antibiotic exposure. Specifically, in line with the data from GF mice, we found a significant decrease in miR-206-3p and miR-219a-2-3p and an increase in miR-369-3p in the amygdala of rats exposed to antibiotics (Fig. 4b–d). Within the PFC of antibiotic-treated rats, we again found a significant decrease in the expression of miR-219a-5p (Fig. 4k), another miRNA found to be differentially expressed in our GF mice; however, the direction of the change was opposite. Other miRNAs that were reported and validated to be altered in GF mice were investigated but did not change following antibiotic exposure in adulthood (Fig. 4e–j, l–m).

## Discussion

A large body of preclinical literature highlights host-microbe interactions as a key factor in the modulation of specific brain networks related to a range of psychiatric disorders [2, 35]. Specifically, using GF animals, many studies have illustrated the critical role of a functional host microbiome in the normal expression of anxiety-, social-, fear- and depressive-like behaviours in these rodents [6, 9, 10, 36] (Additional file 8: Figure S3). Our findings indicated that two brain regions, the PFC and amygdala, known to govern the expression of fear, anxiety and social behaviours, display a large dysregulated network of miRNA expression, which may ultimately contribute to the observed behavioural changes associated with GF mice. We have also shown that exGF mice show an alteration in expression of miRNAs. Complementary approaches using antibiotics offer converging evidence for microbial regulation of miRNA expression. To our knowledge, this is the first time that the gut microbiome has been implicated in miRNA expression in the amygdala and PFC.

Many studies have shown that when GF animals are exposed to microbes early in life, between post-natal weeks 3–6, some of the observed differences can be reversed or partially reversed [6, 8, 37, 38] (Additional file 3: Figure S3). Here, when we allowed for colonization of our GF animals in a conventional facility from post-natal day 21, we found a proportion of miRNAs to have normalized expression levels. Additionally, we found that the majority of differentially regulated miRNAs between CON and GF mice showed regional specificity. These results suggest that targeting the microbiota later in life can have an effect on miRNA expression in the CNS in a brain region-specific manner. We also noted a large number of miRNAs that remained altered following post-weaning exposure to microbes. This supports the concept of critical neurodevelopmental windows during which the gut microbiota is essential in influencing brain development [39]. Previous studies have highlighted that colonization of GF mice with a conventional or reduced microbiota, or by mono-association with just one specific bacterial strain, had the capacity to reverse increased adrenocorticotropic hormone (ACTH) levels after acute stress [37], increased tryptophan levels, reduced basal anxiety [6] and altered social behaviour [8]. It is tempting to speculate that the subset of miRNAs identified that are normalized by colonization may contribute to the well-established phenotype of GF mice as they show dynamic expression, dependent on the presence or absence of a functional microbiome. However, a recent study investigating the changes in hippocampal miRNAs using a hybridized microarray approach in GF and colonized GF mice after weaning showed normalization of seven miRNAs with no restoration of behavioural changes in the open field test [40]. Similarly, we see partial reversibility in miRNAs within the amygdala and PFC but the majority remain altered in our exGF mice. A recent study has demonstrated that colonization of GF mice partially restores impaired amygdala-dependent fear memory recall in GF mice [7]. This partial recovery along with normalization of changes in miRNAs may aid in identification of microbially regulated brain-specific miRNAs. Further studies should continue to investigate more precise behavioural tests that better correlate with specific brain regions.

Within the amygdala, we found that miR-183-5p and miR-182-5p were both decreased and subsequently normalized by colonization. Both of these miRNAs have been previously linked to amygdala-dependent stress- and fear-related outputs [23, 41]. Specifically, miR-183 has been linked to regulating anxiety-related behaviours in the Indian field mouse through influencing acetylcholinesterase splicing [33]. This is also in line with the fact that GF rodents have been shown to display altered basal anxiety levels under naïve conditions and hypersecretion of corticosterone (CORT) under acute stress [37]. Clinically, miR-183 has been shown to be upregulated in whole blood samples from depressed patients on antidepressant treatment [42], highlighting that miR-183 is highly responsive to emotional stimuli. Within the lateral amygdala, miR-182 appears to be essential for long-term amygdala-dependent memory formation assessed by auditory fear conditioning [23]. Recently, it has been revealed that GF animals have impaired fear memory recall [7]. Future studies may aim to manipulate the expression of miRNAs such as miR-182 in order to normalize the amygdala-dependent memory impairments in these mice.

The GF model has many strengths; however, it has limitations in regard to investigating the impact of altered gut microbiota later in life [4, 43–45]. Here, we further validated the lead candidate miRNAs identified in GF mice by following alternative microbiota manipulation strategy in a different species. We used brain tissue from a separate cohort of rats exposed to antibiotics during adulthood following normal development of the gut microbiota prior to weaning [30]. Behavioural assessment of these rodents post microbiota depletion showed impairments in cognition and induced depressive-like behaviours [30]. We found that alterations in miR-219a-2-3p expression in both the amygdala and PFC was a common feature of both GF status and animals rendered microbiota-deficient post-weaning by antibiotic exposure. Sequencing-based studies have found miR-219a-2-3p/miR-219-3p to be altered in the basolateral amygdala following social defeat [46]. Thus, even when the gut microbiota is present during early life, subsequent depletion via antibiotics still indicates a role of gut microbiota in miRNA regulation. Whether gut microbiota-directed interventions that produce more qualitative differences in the microbiome also differentially regulate miRNAs is an open question. Colonization of microbiota-deficient rodents with the microbiota from mice with different behavioural phenotypes, as described by Bercik et al., or incremental antibiotic doses, would also provide valuable insights [47]. Additionally, direct strain comparison between GF and antibiotic-treated mice may better reveal brain-specific miRNAs sensitive to changes in the gut microbiota.

Both strategies employed in our study to investigate the effect of the gut microbiota on miRNA expression implicated miR-206-3p as a target of the gut microbiota, which was decreased under both conditions (Figs. 2g and 4b). This particular miRNA is well validated in regard to its role in the regulation of BDNF, an essential neurotrophin, which promotes growth and development of new neurons, survival of existing ones, and has an essential role in synaptic plasticity [48]. Altered BDNF expression is a hallmark of a disturbed microbiota-gut-brain axis, and it has been repeatedly shown to be altered in GF and antibiotic-treated rodents [18, 27, 49, 50]. BDNF mRNAs are proposed to contain conserved binding sites for at least 14 different miRNA families with many predicted interactions validated in vivo [51]. Specifically, miR-206-3p has been implicated in regulating the levels of BDNF in different animal models of neurological disorders [51, 52]. We have previously shown that there is a significant increase in the mRNA levels of a specific BDNF transcript [27] in the amygdala, consistent with the finding that miR-206-3p is significantly downregulated in this region in the current study. Additionally, a decrease in miR-206-3p in our antibiotic-treated rats coincided with a decrease in BDNF mRNA [30]. Previous work has revealed that targeting BDNF signalling by miRNAs has therapeutic potential in neurodegenerative and psychiatric diseases [48]. Thus, BDNF levels may be regulated by microbiota-induced alterations in miRNA expression and thus holds potential for a new treatment strategy in many CNS-related disorders.

Our analysis of predicted mRNA targets reveals KEGG pathways enriched for neurotrophin signalling along with a prominent representation of targets enriched in GO terms for nervous system development, neurogenesis, neuronal development and differentiation. miRNA dysregulation and/or dysfunction are believed to be underlying factors contributing to neurodegenerative diseases and neurodevelopmental abnormalities [53]. In fact, GF status results in altered stress circuitry along with several well-documented behavioural alterations, cognitive impairment, impaired microglial activation and abnormally altered myelination [4]. A recent study in GF mice demonstrated that within the amygdala, there is significant volumetric expansion within the lateral, basolateral and medial nucleus when compared to CON mice [19]. Coinciding with this, GF mice display hypertrophy of aspiny interneurons and pyramidal neurons along with changes in spine density [19].

Whether this change in gross morphology of the amygdala in adulthood is related to the large change in miRNAs remains to be determined. However, linking changes in miRNAs to changes in amygdala volume has been demonstrated in a rat model of autism using valproic acid. Administration of valproic acid coincides with an enlarged amygdala and increased miR-30d and miR-181c (~ 1.2 fold increase). Functional enrichment of predicted targets of both miRNAs indicates enrichment in GO terms for tissue morphology, nervous system development and cellular development, which was confirmed by in vitro inhibition of miR-181c [54]. We also see a trend towards a significant increase in miR-181c-5p (1.4 fold increase) in our sequencing data (Additional file 1: Table S1), which may be contributing to the gross morphological changes seen in GF mice.

The exact mechanism through which the gut microbiota can influence the expression of non-coding RNAs such as miRNAs remains unclear. Currently, most evidence supports signalling through the vagus nerve [14, 55] and bacterial metabolites such as short-chain fatty acids (SCFAs), which indirectly affect the nervous system through their immunomodulatory functions [56, 57]. Recently, a study investigating the impact of the gut microbiota on myelination demonstrated that certain bacterial metabolites in vitro can impair oligodendrocyte differentiation [58].

## Conclusion

In conclusion, the present study indicates that appropriate regulation of miRNA expression within the amygdala and PFC is influenced by microbiota composition and activity and relies on the presence of a functional microbiota during critical windows of neurodevelopment. In-depth analysis of mRNA targets predicted to be under the influence of noted dysregulated miRNAs further suggests these miRNAs may be implicated in neuronal development, neurogenesis and appropriate BDNF signalling, all of which have been shown to be altered in GF mice. Our results further highlight that even following normal gut microbiome development, subsequent knockdown with antibiotics also impacts CNS miRNA expression and that such effects generalize to another species (rat). The information garnered from these two commonly applied strategies particularly highlight miR-206-3p, a well-characterized miRNA that is essential in the regulation of BDNF. Further mono-association studies may elucidate specific bacterial strains that mediate the influence over miRNAs, which would considerably aid our efforts to fully delineate how certain bacteria influence CNS function. miRNA-based therapeutics for fear and anxiety disorders face a number of obstacles relating to the availability of safe compounds that are able to cross the blood-brain barrier and target relevant cell populations and neurocircuitry [59]. Coupled with the recent data supporting a role for the microbiota in amygdala-dependent fear recall, the possibility of achieving the desired impact on miRNA expression in specific brain regions by targeting the gut microbiota is an appealing prospect. This may expedite the promise apparent in these two previously disparate approaches.

## Additional files

Additional file 1: Table S1. Differentially regulated microRNAs in the PFC and amygdala of GF and exGF mice. (XLSX 42 kb)

Additional file 2: Table S2. Probe assay IDs for individual miRNAs used in validation experiments. (PPTX 36 kb)

Additional file 3: Figure S1. Venn diagrams representing the number of up- and downregulated miRNAs. All miRNAs in the amygdala (a, b) and PFC (c, d) across all group comparisons (CON vs. GF, GF vs. exGF and CON vs. exGF) that are differentially regulated. Red circle represents one miRNA where its expression levels were increased even further than in GF post colonisation. (PPTX 117 kb)

Additional file 4: Table S3. Selected miRNAs for validation via qRT-PCR in GF mice. Table represents all miRNAs that were reported to be significantly altered in GF mice with accompanying mature miRNA sequence, whether they are conserved across mice, rats and humans and the number of conserved mRNA targets for that miRNA as predicted by miRwalk. M/R (mouse/rat). (PPTX 40 kb)

Additional file 5: Figure S2. Functional enrichment analysis of predicted mRNA targets of differentially regulated miRNAs in the PFC. (a) Number of miRNA (out of 9) where its predicted targets are enriched for GO terms. (b) KEGG pathway that are predicted to be enriched in the amygdala based on the mRNA targets of all differentially regulated miRNAs in GF mice. (c) Venn diagrams depicting overlaps in enriched GO terms and KEGG pathways between the amygdala and PFC. Bar graphs depict the number of miRNA that have predicted mRNA targets that fall into specific GO terms and KEGG pathways. Scatter plot depicts how significant individual miRNAs are enriched for a specific GO term or KEGG pathway. (PPTX 210 kb)

Additional file 6: Table S4. miRNA/mRNA predicted interaction and overlap with mRNA sequencing in the amygdala of GF mice. List of all qRT-PCR validated miRNAs in the amygdala that are predicted to target mRNAs that are dysregulated in GF mice. This table is based on comparison between CON vs GF mice. Dysregulated genes (DEGs). (PPTX 39 kb)

Additional file 7: Table S5. miRNA/mRNA predicted interaction and overlap with mRNA sequencing in the PFC of GF mice. List of all miRNAs that are downregulated as indicated by Illumina sequencing in the PFC that are predicted to target myelin-related genes that are increased in the PFC of GF mice. This table is based on comparison between CON vs GF mice. Dysregulated genes (DEGs). (PPTX 34 kb)

Additional file 8: Figure S3. The behavioural phenotype of GF animals features abnormalities in behaviours controlled by the amygdala and PFC. (A) Percentage control transitions in the light-dark box [6]. (b) Total percentage freezing during fear extinction [7]. (c) Percent time graphed as % control mean ± SEM *P* < 0.05* and *P* < 0.01**. (PPTX 266 kb)

## Abbreviations

BDNF: Brain-derived neurotrophic factor
CON: Conventional
exGF: Germ-free colonized
GF: Germ-free
GO: Gene Ontology
KEGG: Kyoto Encyclopedia of Genes and Genomes
miRNA: MicroRNA
NGS: Next-generation sequencing
PFC: Prefrontal cortex
SCFAs: Short-chain fatty acids
TPM: Tags per million
