# Supplementary material for: Microbial regulation of microRNA expression in the amygdala and prefrontal cortex
Source: Microbiome. 2017 Aug 25;5:102. doi: 10.1186/s40168-017-0321-3 (PMC5571609; doi:10.1186/s40168-017-0321-3)
Supplement: Supplementary file 2 — Probe assay IDs for individual miRNAs used in validation experiments. (PPTX 36 kb) [file 40168_2017_321_MOESM2_ESM.pptx]

## Slide 1
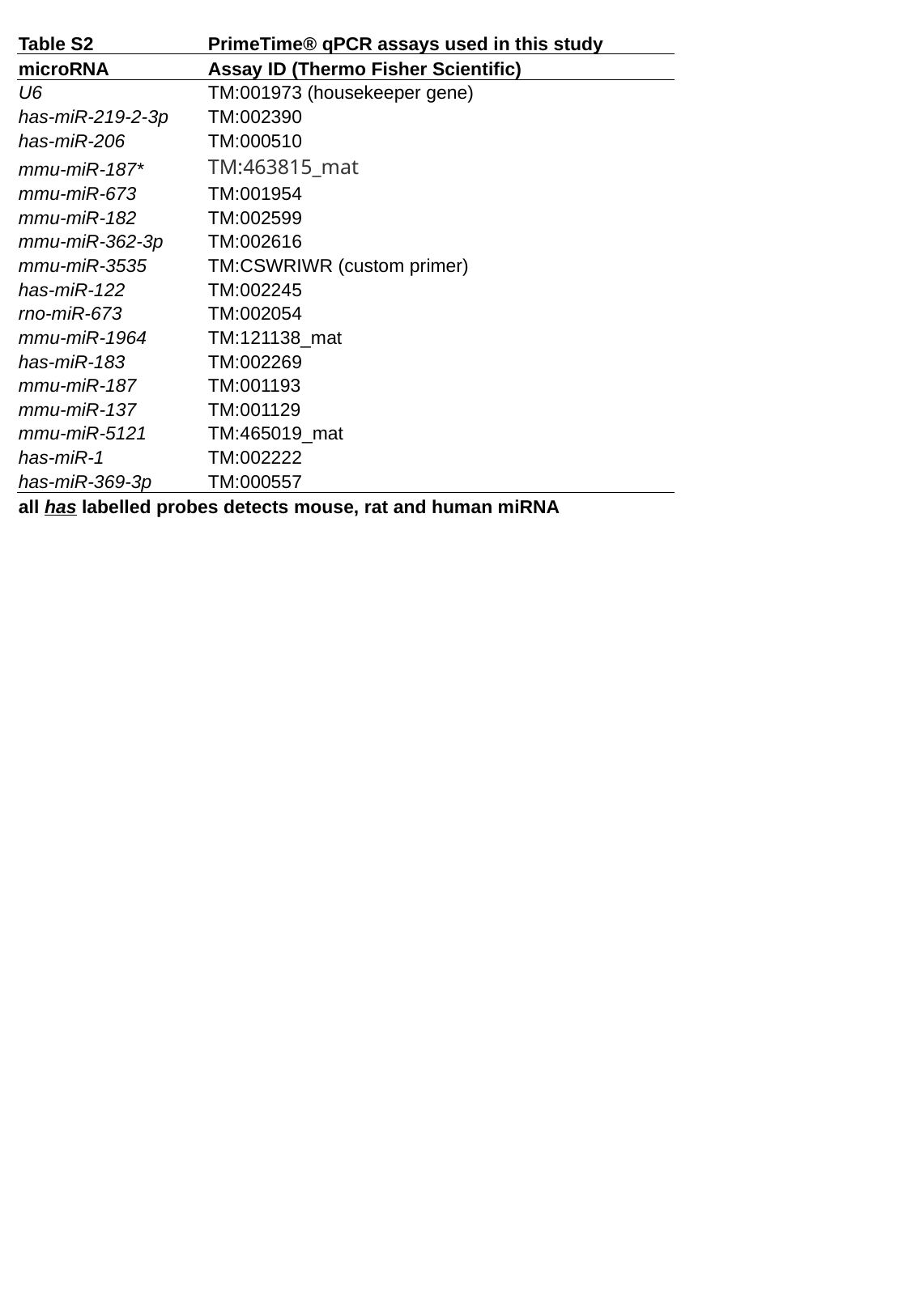

| Table S2 | PrimeTime® qPCR assays used in this study |
| --- | --- |
| microRNA | Assay ID (Thermo Fisher Scientific) |
| U6 | TM:001973 (housekeeper gene) |
| has-miR-219-2-3p | TM:002390 |
| has-miR-206 | TM:000510 |
| mmu-miR-187\* | TM:463815\_mat |
| mmu-miR-673 | TM:001954 |
| mmu-miR-182 | TM:002599 |
| mmu-miR-362-3p | TM:002616 |
| mmu-miR-3535 | TM:CSWRIWR (custom primer) |
| has-miR-122 | TM:002245 |
| rno-miR-673 | TM:002054 |
| mmu-miR-1964 | TM:121138\_mat |
| has-miR-183 | TM:002269 |
| mmu-miR-187 | TM:001193 |
| mmu-miR-137 | TM:001129 |
| mmu-miR-5121 | TM:465019\_mat |
| has-miR-1 | TM:002222 |
| has-miR-369-3p | TM:000557 |
| all has labelled probes detects mouse, rat and human miRNA | |
