# Supplementary material for: Microbial regulation of microRNA expression in the amygdala and prefrontal cortex
Source: Microbiome. 2017 Aug 25;5:102. doi: 10.1186/s40168-017-0321-3 (PMC5571609; doi:10.1186/s40168-017-0321-3)
Supplement: Supplementary file 3 — Venn diagrams representing the number of up- and downregulated miRNAs. All miRNAs in the amygdala (a, b) and PFC (c, d) across all group comparisons (CON vs. GF, GF vs. exGF and CON vs. exGF) that are differentially regulated. Red circle represents one miRNA where its expression levels were increased even further than in GF post colonisation. (PPTX 117 kb) [file 40168_2017_321_MOESM3_ESM.pptx]

## Slide 1
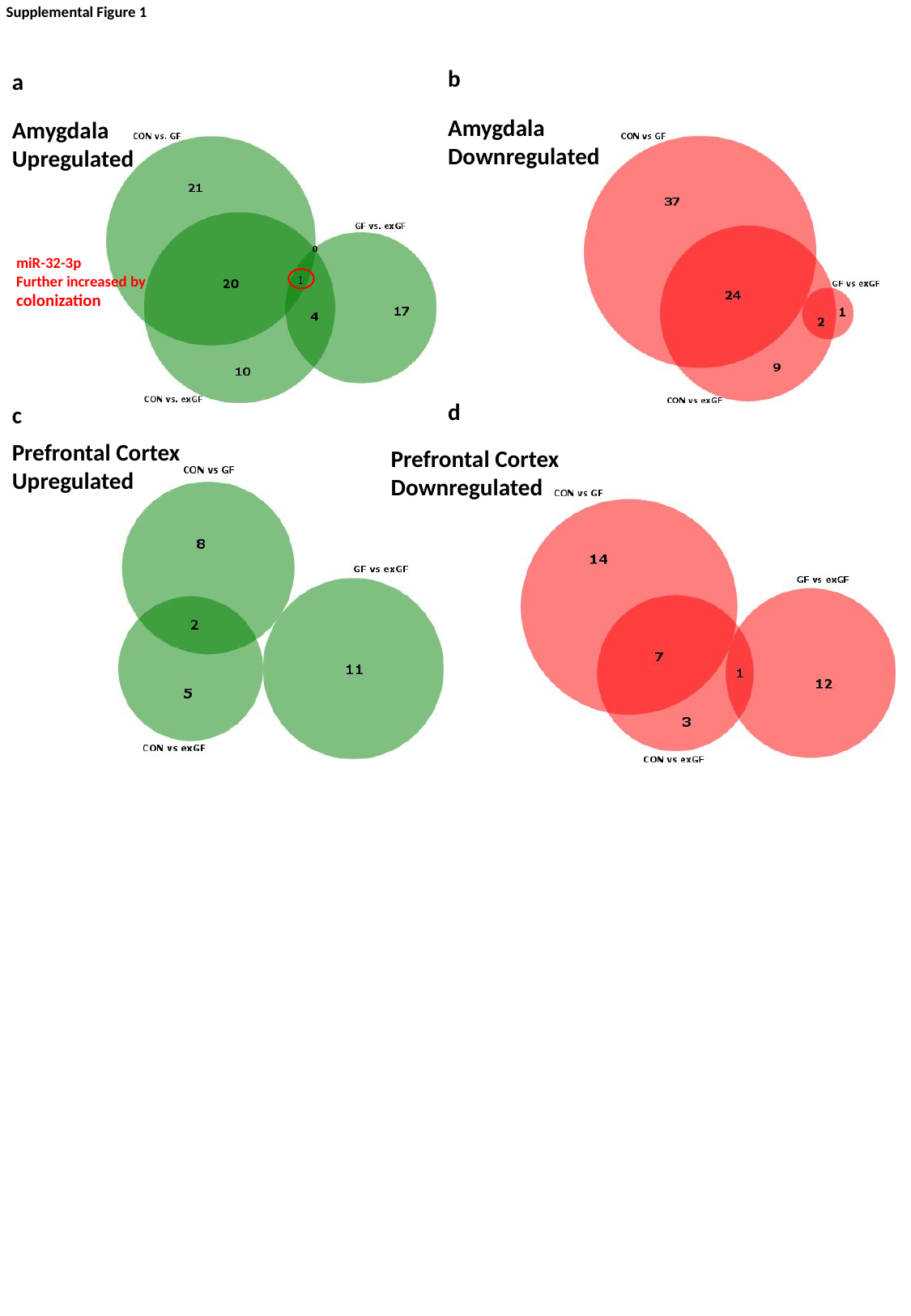

Supplemental Figure 1
b
a
Amygdala
Downregulated
Amygdala
Upregulated
miR-32-3p
Further increased by colonization
d
c
Prefrontal Cortex
Upregulated
Prefrontal Cortex
Downregulated
