# Supplementary material for: Microbial regulation of microRNA expression in the amygdala and prefrontal cortex
Source: Microbiome. 2017 Aug 25;5:102. doi: 10.1186/s40168-017-0321-3 (PMC5571609; doi:10.1186/s40168-017-0321-3)
Supplement: Supplementary file 4 — Selected miRNAs for validation via qRT-PCR in GF mice. Table represents all miRNAs that were reported to be significantly altered in GF mice with accompanying mature miRNA sequence, whether they are conserved across mice, rats and humans and the number of conserved mRNA targets for that miRNA as predicted by miRwalk. M/R (mouse/rat). (PPTX 40 kb) [file 40168_2017_321_MOESM4_ESM.pptx]

## Slide 1
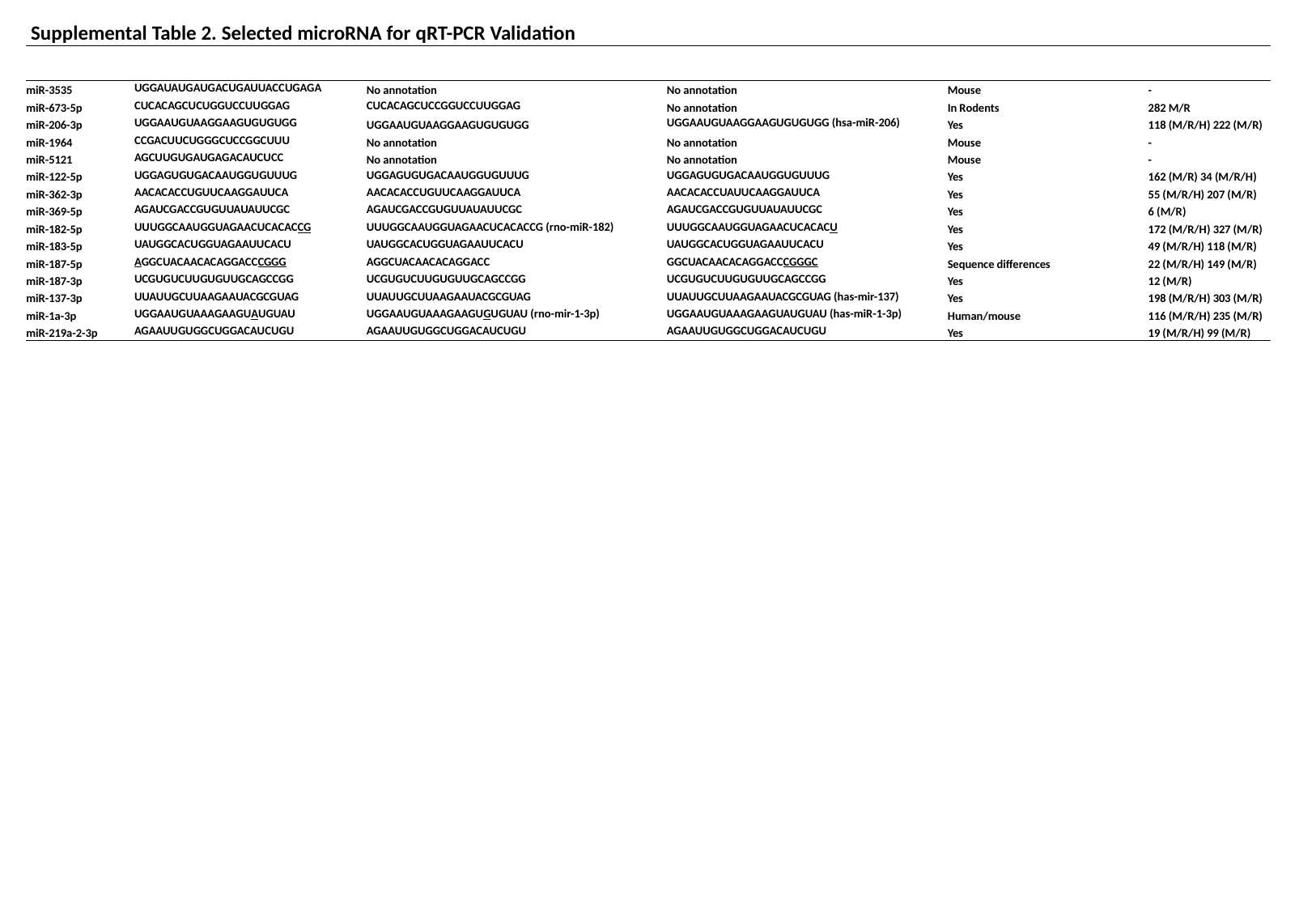

Supplemental Table 2. Selected microRNA for qRT-PCR Validation
| | | | | | |
| --- | --- | --- | --- | --- | --- |
| Mature MicroRNA | Mouse Sequence | Rat Sequence | Human Sequence | Conserved MicroRNA? | No. of conserved mRNA targets |
| miR-3535 | UGGAUAUGAUGACUGAUUACCUGAGA | No annotation | No annotation | Mouse | - |
| miR-673-5p | CUCACAGCUCUGGUCCUUGGAG | CUCACAGCUCCGGUCCUUGGAG | No annotation | In Rodents | 282 M/R |
| miR-206-3p | UGGAAUGUAAGGAAGUGUGUGG | UGGAAUGUAAGGAAGUGUGUGG | UGGAAUGUAAGGAAGUGUGUGG (hsa-miR-206) | Yes | 118 (M/R/H) 222 (M/R) |
| miR-1964 | CCGACUUCUGGGCUCCGGCUUU | No annotation | No annotation | Mouse | - |
| miR-5121 | AGCUUGUGAUGAGACAUCUCC | No annotation | No annotation | Mouse | - |
| miR-122-5p | UGGAGUGUGACAAUGGUGUUUG | UGGAGUGUGACAAUGGUGUUUG | UGGAGUGUGACAAUGGUGUUUG | Yes | 162 (M/R) 34 (M/R/H) |
| miR-362-3p | AACACACCUGUUCAAGGAUUCA | AACACACCUGUUCAAGGAUUCA | AACACACCUAUUCAAGGAUUCA | Yes | 55 (M/R/H) 207 (M/R) |
| miR-369-5p | AGAUCGACCGUGUUAUAUUCGC | AGAUCGACCGUGUUAUAUUCGC | AGAUCGACCGUGUUAUAUUCGC | Yes | 6 (M/R) |
| miR-182-5p | UUUGGCAAUGGUAGAACUCACACCG | UUUGGCAAUGGUAGAACUCACACCG (rno-miR-182) | UUUGGCAAUGGUAGAACUCACACU | Yes | 172 (M/R/H) 327 (M/R) |
| miR-183-5p | UAUGGCACUGGUAGAAUUCACU | UAUGGCACUGGUAGAAUUCACU | UAUGGCACUGGUAGAAUUCACU | Yes | 49 (M/R/H) 118 (M/R) |
| miR-187-5p | AGGCUACAACACAGGACCCGGG | AGGCUACAACACAGGACC | GGCUACAACACAGGACCCGGGC | Sequence differences | 22 (M/R/H) 149 (M/R) |
| miR-187-3p | UCGUGUCUUGUGUUGCAGCCGG | UCGUGUCUUGUGUUGCAGCCGG | UCGUGUCUUGUGUUGCAGCCGG | Yes | 12 (M/R) |
| miR-137-3p | UUAUUGCUUAAGAAUACGCGUAG | UUAUUGCUUAAGAAUACGCGUAG | UUAUUGCUUAAGAAUACGCGUAG (has-mir-137) | Yes | 198 (M/R/H) 303 (M/R) |
| miR-1a-3p | UGGAAUGUAAAGAAGUAUGUAU | UGGAAUGUAAAGAAGUGUGUAU (rno-mir-1-3p) | UGGAAUGUAAAGAAGUAUGUAU (has-miR-1-3p) | Human/mouse | 116 (M/R/H) 235 (M/R) |
| miR-219a-2-3p | AGAAUUGUGGCUGGACAUCUGU | AGAAUUGUGGCUGGACAUCUGU | AGAAUUGUGGCUGGACAUCUGU | Yes | 19 (M/R/H) 99 (M/R) |
| | | | | | |
