# Supplementary material for: Microbial regulation of microRNA expression in the amygdala and prefrontal cortex
Source: Microbiome. 2017 Aug 25;5:102. doi: 10.1186/s40168-017-0321-3 (PMC5571609; doi:10.1186/s40168-017-0321-3)
Supplement: Supplementary file 5 — Functional enrichment analysis of predicted mRNA targets of differentially regulated miRNAs in the PFC. (a) Number of miRNA (out of 9) where its predicted targets are enriched for GO terms. (b) KEGG pathway that are predicted to be enriched in the amygdala based on the mRNA targets of all differentially regulated miRNAs in GF mice. (c) Venn diagrams depicting overlaps in enriched GO terms and KEGG pathways between the amygdala and PFC. Bar graphs depict the number of miRNA that have predicted mRNA targets that fall into specific GO terms and KEGG pathways. Scatter plot depicts how significant individual miRNAs are enriched for a specific GO term or KEGG pathway. (PPTX 210 kb) [file 40168_2017_321_MOESM5_ESM.pptx]

## Slide 1
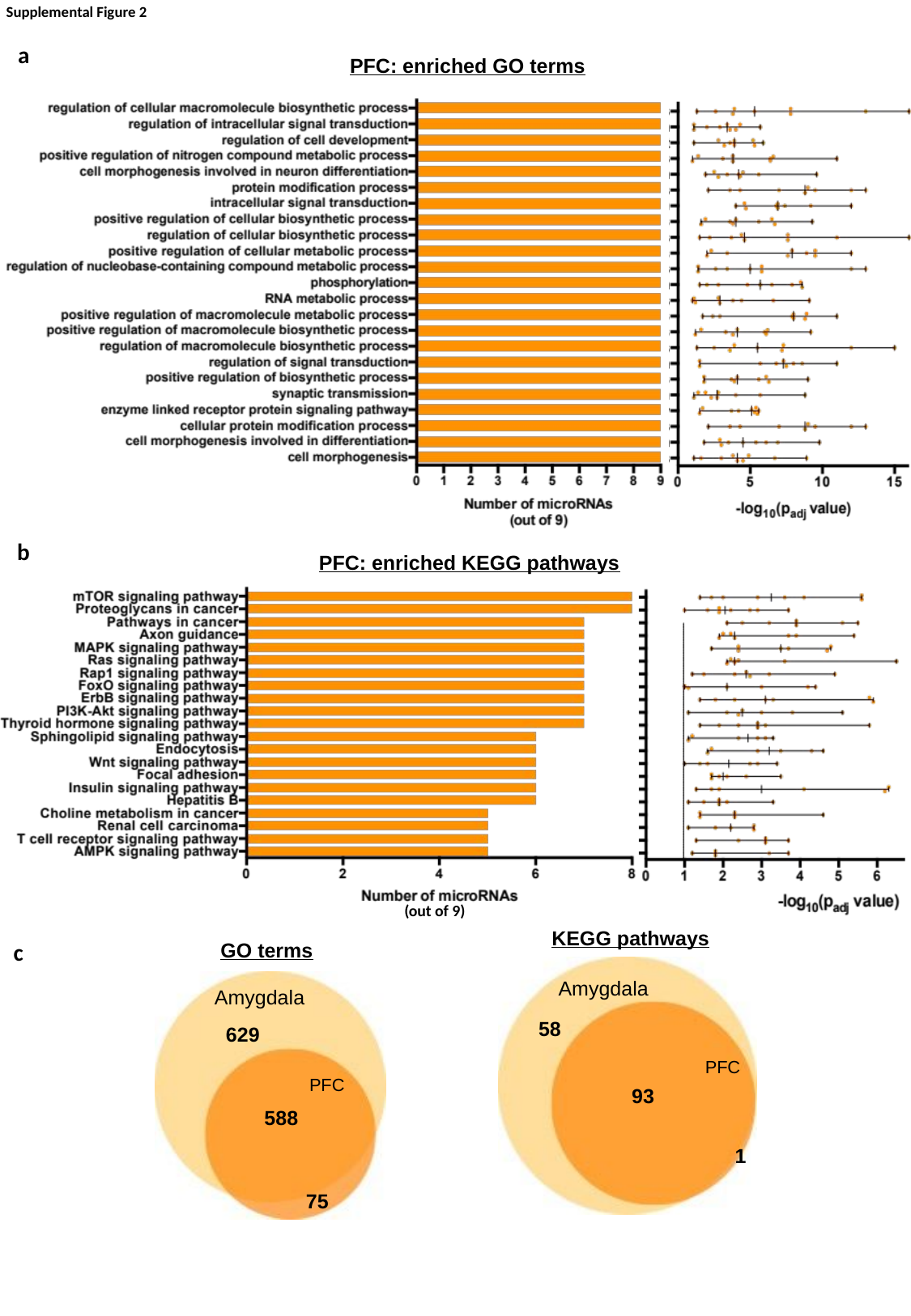

Supplemental Figure 2
a
PFC: enriched GO terms
b
PFC: enriched KEGG pathways
(out of 9)
KEGG pathways
c
GO terms
Amygdala
Amygdala
58
629
PFC
PFC
93
588
1
75
