# Supplementary material for: Microbial regulation of microRNA expression in the amygdala and prefrontal cortex
Source: Microbiome. 2017 Aug 25;5:102. doi: 10.1186/s40168-017-0321-3 (PMC5571609; doi:10.1186/s40168-017-0321-3)
Supplement: Supplementary file 6 — miRNA/mRNA predicted interaction and overlap with mRNA sequencing in the amygdala of GF mice. List of all qRT-PCR validated miRNAs in the amygdala that are predicted to target mRNAs that are dysregulated in GF mice. This table is based on comparison between CON vs GF mice. Dysregulated genes (DEGs). (PPTX 39 kb) [file 40168_2017_321_MOESM6_ESM.pptx]

## Slide 1
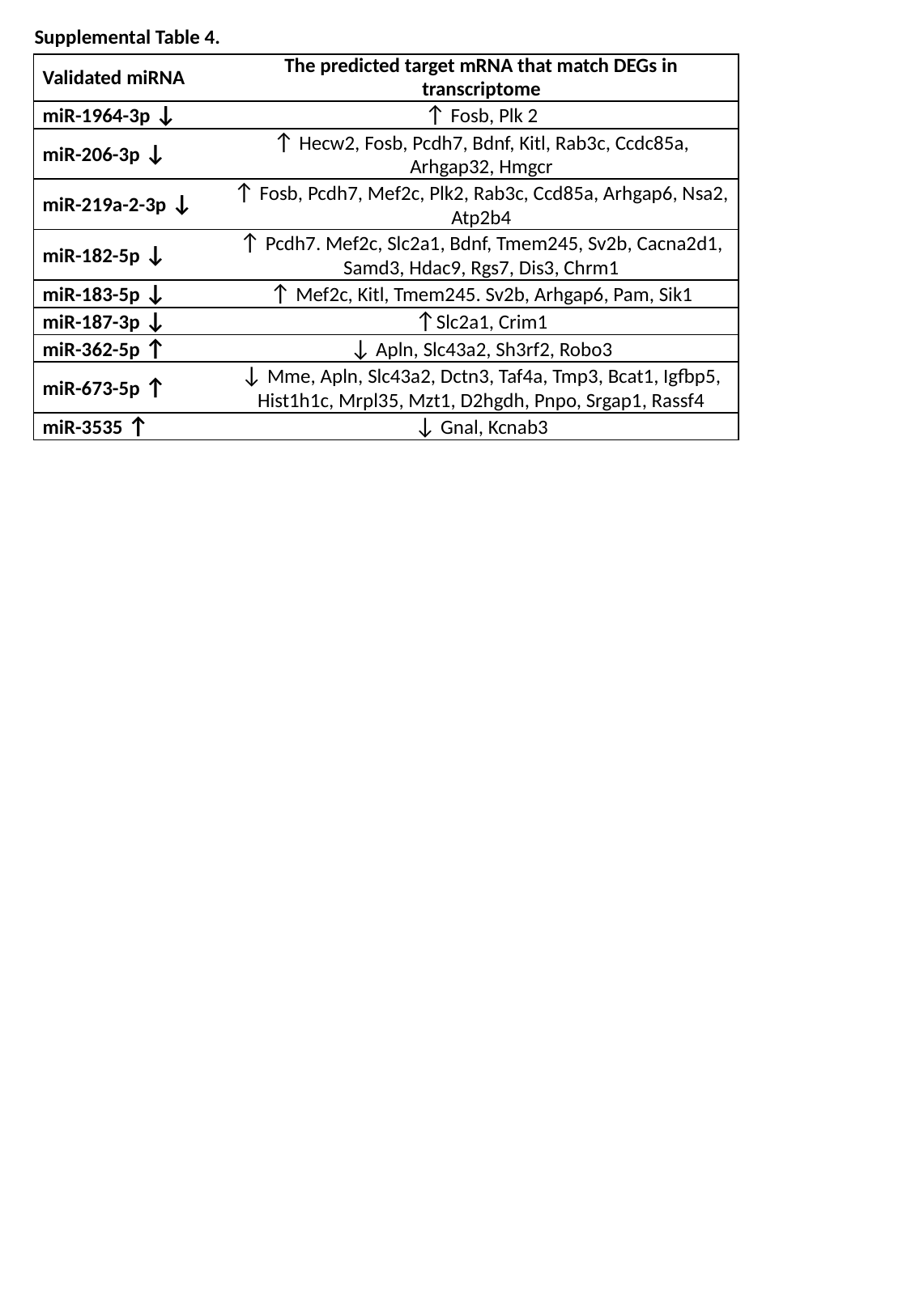

Supplemental Table 4.
| Validated miRNA | The predicted target mRNA that match DEGs in transcriptome |
| --- | --- |
| miR-1964-3p ↓ | ↑ Fosb, Plk 2 |
| miR-206-3p ↓ | ↑ Hecw2, Fosb, Pcdh7, Bdnf, Kitl, Rab3c, Ccdc85a, Arhgap32, Hmgcr |
| miR-219a-2-3p ↓ | ↑ Fosb, Pcdh7, Mef2c, Plk2, Rab3c, Ccd85a, Arhgap6, Nsa2, Atp2b4 |
| miR-182-5p ↓ | ↑ Pcdh7. Mef2c, Slc2a1, Bdnf, Tmem245, Sv2b, Cacna2d1, Samd3, Hdac9, Rgs7, Dis3, Chrm1 |
| miR-183-5p ↓ | ↑ Mef2c, Kitl, Tmem245. Sv2b, Arhgap6, Pam, Sik1 |
| miR-187-3p ↓ | ↑Slc2a1, Crim1 |
| miR-362-5p ↑ | ↓ Apln, Slc43a2, Sh3rf2, Robo3 |
| miR-673-5p ↑ | ↓ Mme, Apln, Slc43a2, Dctn3, Taf4a, Tmp3, Bcat1, Igfbp5, Hist1h1c, Mrpl35, Mzt1, D2hgdh, Pnpo, Srgap1, Rassf4 |
| miR-3535 ↑ | ↓ Gnal, Kcnab3 |
