# Supplementary material for: Microbial regulation of microRNA expression in the amygdala and prefrontal cortex
Source: Microbiome. 2017 Aug 25;5:102. doi: 10.1186/s40168-017-0321-3 (PMC5571609; doi:10.1186/s40168-017-0321-3)
Supplement: Supplementary file 7 — miRNA/mRNA predicted interaction and overlap with mRNA sequencing in the PFC of GF mice. List of all miRNAs that are downregulated as indicated by Illumina sequencing in the PFC that are predicted to target myelin-related genes that are increased in the PFC of GF mice. This table is based on comparison between CON vs GF mice. Dysregulated genes (DEGs). (PPTX 34 kb) [file 40168_2017_321_MOESM7_ESM.pptx]

## Slide 1
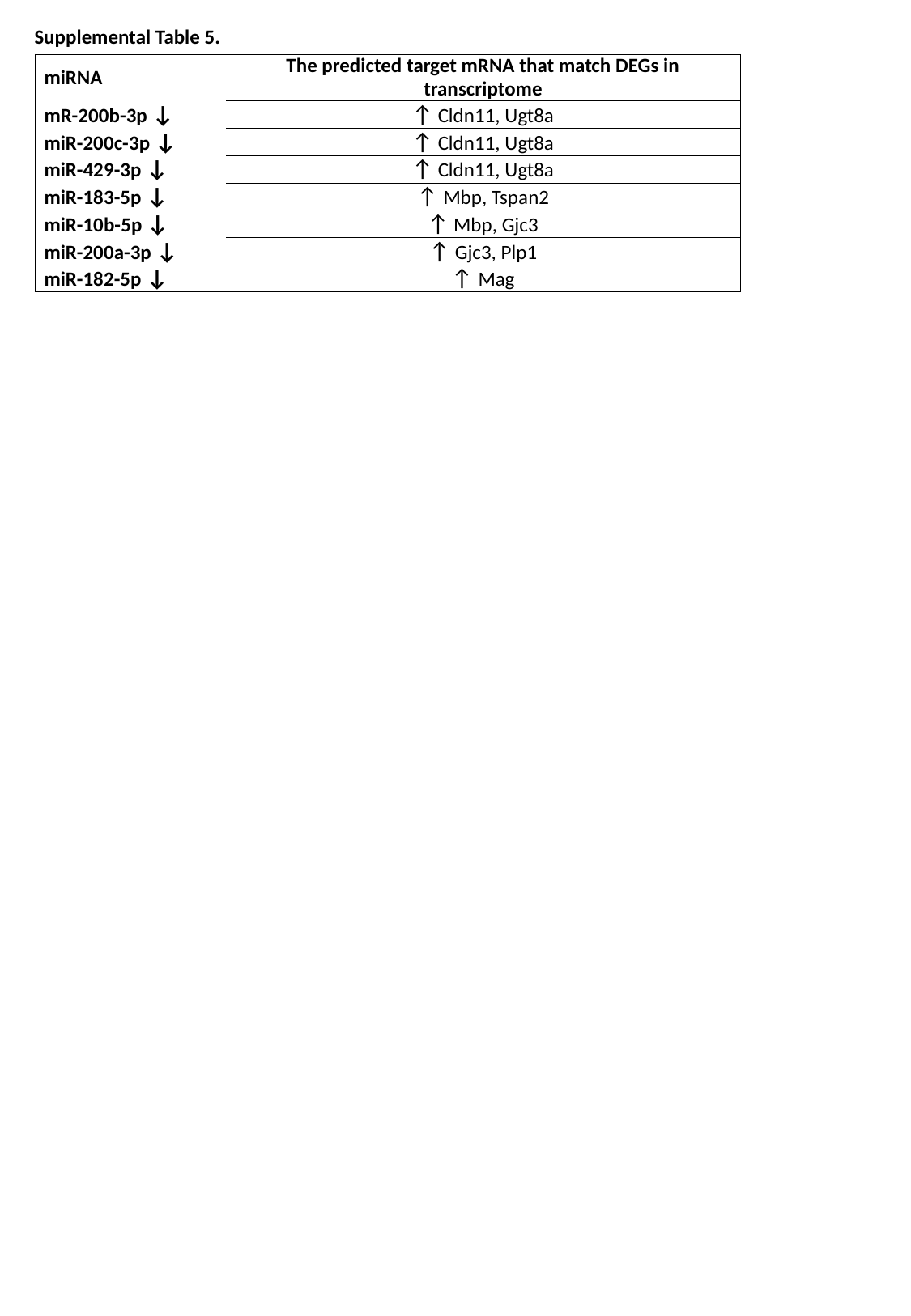

Supplemental Table 5.
| miRNA | The predicted target mRNA that match DEGs in transcriptome |
| --- | --- |
| mR-200b-3p ↓ | ↑ Cldn11, Ugt8a |
| miR-200c-3p ↓ | ↑ Cldn11, Ugt8a |
| miR-429-3p ↓ | ↑ Cldn11, Ugt8a |
| miR-183-5p ↓ | ↑ Mbp, Tspan2 |
| miR-10b-5p ↓ | ↑ Mbp, Gjc3 |
| miR-200a-3p ↓ | ↑ Gjc3, Plp1 |
| miR-182-5p ↓ | ↑ Mag |
