# Supplementary material for: Microbial regulation of microRNA expression in the amygdala and prefrontal cortex
Source: Microbiome. 2017 Aug 25;5:102. doi: 10.1186/s40168-017-0321-3 (PMC5571609; doi:10.1186/s40168-017-0321-3)
Supplement: Supplementary file 8 — The behavioural phenotype of GF animals features abnormalities in behaviours controlled by the amygdala and PFC. (A) Percentage control transitions in the light-dark box [6]. (b) Total percentage freezing during fear extinction [7]. (c) Percent time graphed as % control mean ± SEM P < 0.05* and P < 0.01**. (PPTX 266 kb) [file 40168_2017_321_MOESM8_ESM.pptx]

## Slide 1
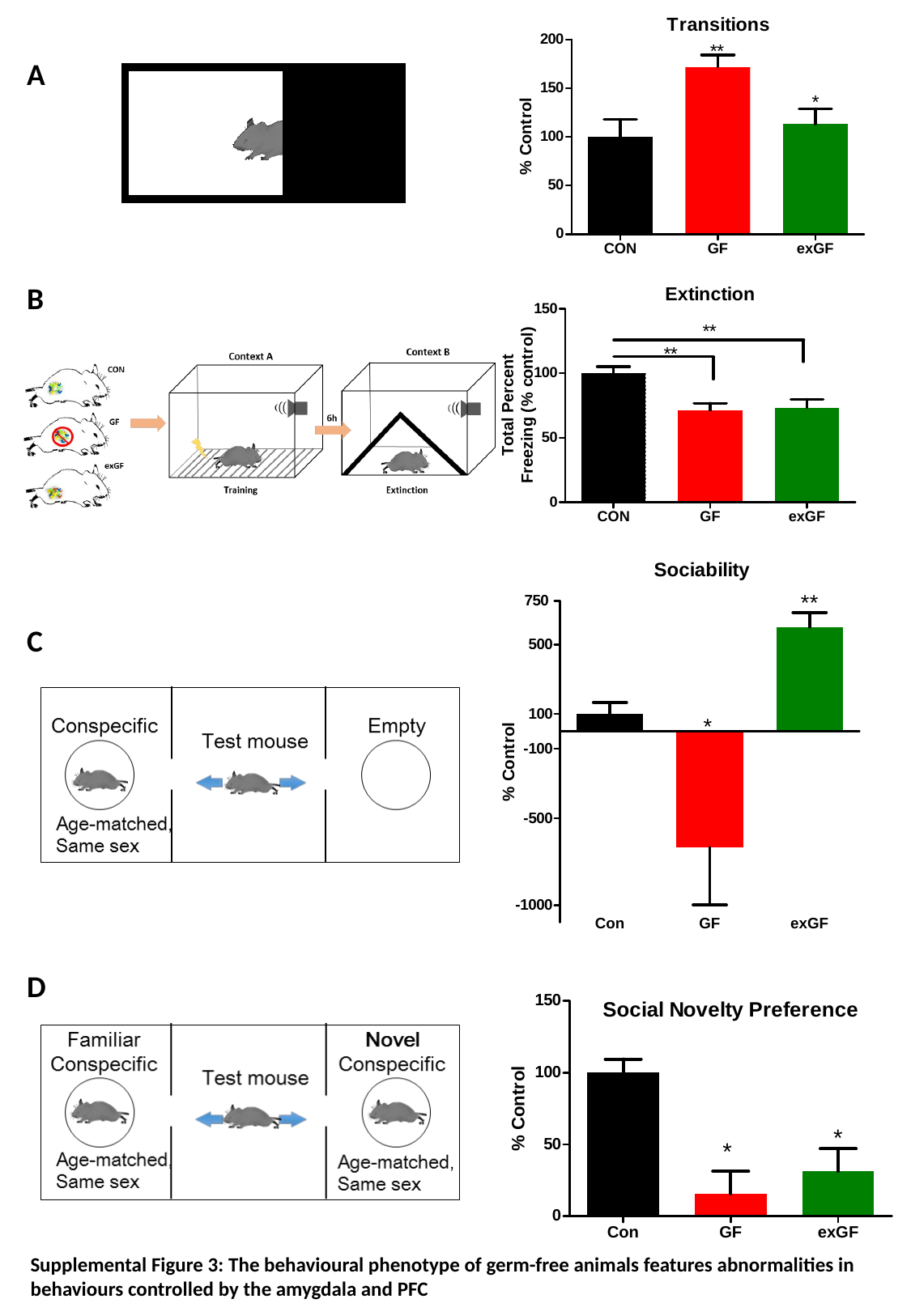

A
B
C
D
Supplemental Figure 3: The behavioural phenotype of germ-free animals features abnormalities in behaviours controlled by the amygdala and PFC
